# Methamphetamine Use in People Living with HIV: Clinical, Neurocognitive, and Blood Biomarker Profiles

**DOI:** 10.3390/biomedicines14020443

**Published:** 2026-02-16

**Authors:** Monserrat Alvarez-Zavala, Nadia I. Álvarez-Álvarez, Jocelyn A. Cabrales-Lozano, Víctor Rodríguez-Pérez, José L. Ruíz-Sandoval, Andrea Torres-Rojas, Adriana Aguayo-Arelis, Tania E. Holguin-Aguirre, Luz A. González-Hernández, Jaime F. Andrade-Villanueva, Fernando Amador-Lara

**Affiliations:** 1 Departamento de Clínicas Médicas, Centro Universitario de Ciencias de la Salud, Universidad de Guadalajara, Guadalajara 44280, Mexico; monse_belan@hotmail.com (M.A.-Z.); luceroga08@gmail.com (L.A.G.-H.); drjandradev@gmail.com (J.F.A.-V.); 2 Unidad de VIH, Hospital Civil de Guadalajara Fray Antonio Alcalde, Guadalajara 44280, Mexico; jocelyn.cabrales@academicos.udg.mx (J.A.C.-L.); taniaholguinaguirre@gmail.com (T.E.H.-A.); 3 Instituto de Investigación en Inmunodeficiencias y VIH, Centro Universitario de Ciencias de la Salud, Universidad de Guadalajara, Hospital 278, Guadalajara 44280, Mexico; andrea.torres2288@alumnos.udg.mx; 4 Departamento de Neurociencias, Maestría en Neurociencias de las Adicciones, Centro Universitario de Ciencias de la Salud, Universidad de Guadalajara, Guadalajara 44340, Mexico; nadia.alvarez9925@alumnos.udg.mx; 5 Departamento de Clínicas de Salud Mental, Centro Universitario de Ciencias de la Salud, Universidad de Guadalajara, Guadalajara 44340, Mexico; 6 Dirección de Prevención y Participación Social, Centro Nacional para la Prevención y Control del VIH y Hepatitis (CENSIDA), Ciudad de Mexico 11570, Mexico; victor.psicologoacademico@gmail.com; 7 Servicio de Neurología, Hospital Civil de Guadalajara Fray Antonio Alcalde, Guadalajara 44280, Mexico; jose.rsandoval@academicos.udg.mx; 8 Programa de Doctorado en Ciencias en Biología Molecular, Centro Universitario de Ciencias de la Salud, Universidad de Guadalajara, Guadalajara 44340, Mexico; 9 Departamento de Psicología Aplicada, Centro Universitario de Ciencias de la Salud, Universidad de Guadalajara, Guadalajara 44340, Mexico; adriana.aguayo@academicos.udg.mx; 10 Programa de VIH, ITS y VHC, OPD Servicios de Salud Jalisco, Guadalajara 44100, Mexico

**Keywords:** methamphetamine use, cognitive performance, HIV infection, biomarkers, sCD14, neuron-specific enolase (NSE), chemsex, neuronal injury, neuroinflammation

## Abstract

**Background:** Methamphetamine (MA) use in people living with HIV (PLWH) has been linked to neurocognitive and behavioral dysregulation. We hypothesized that PLWH with active MA use (MAHIV) would show poorer cognitive performance, greater emotional and sleep burden, higher behavioral risk, and alterations in circulating biomarkers of immune activation and neuronal injury, relative to PLWH without MA use and HIV-negative Controls. **Methods:** Cross-sectional analytic study of 121 adults: PLWH with MA use (MAHIV, *n* = 40), PLWH without use (*n* = 42), and HIV-negative Controls (*n* = 39). Outcomes were ART discontinuation, physical activity, neurocognition (MoCA), depression (BDI), anxiety (GAD-7), sleep (PSQI), and substance use (ASSIST). Circulating biomarkers measured by ELISA: sCD14, neuron-specific enolase (NSE), S100B, and neurofilament light chain (NfL). **Results:** MAHIV participants had more frequent ART discontinuation than PLWH and the lowest physical activity. Chemsex with polysubstance use, condomless sex, and multiple partners were most prevalent in MAHIV. This group showed the highest anxiety and depressive burdens, and the greatest sleep disturbances. Global cognition (MoCA) was lowest in MAHIV, with significant deficits in executive function, memory, attention, and language; 82.5% had at least mild cognitive impairment. sCD14 was significantly higher in MAHIV than in PLWH and Controls, and NSE was elevated in both MAHIV and PLWH versus Controls. sCD14 correlated inversely with MoCA and positively with GAD-7 and BDI-II. **Conclusions:** Among PLWH, MA use is associated with greater ART nonadherence, syndemic mental-health and sleep disturbances, broader neurocognitive deficits, and elevations in circulating sCD14 and NSE. The sCD14–cognition and sCD14–mood relationships highlight chronic immune activation as a candidate pathway for neurocognitive and affective impairment and support sCD14 and NSE as potential stratification and monitoring biomarkers in MA-using PLWH.

## 1. Introduction

Methamphetamine (MA) is a potent psychostimulant and neurotoxic drug of abuse with a rapidly expanding global footprint. Current estimates indicate that more than 30 million individuals use amphetamine-type stimulants, including MA, and multiple regions have documented a marked resurgence in MA consumption over the past decade. The social consequences of MA use (job loss, family disruption, legal problems, financial instability) underscore the urgency of scalable diagnostic and therapeutic strategies. Importantly, MA use is disproportionately prevalent in certain populations, particularly among people living with HIV (PLWH), in whom it has been linked to worse clinical outcomes [1]. In some urban cohorts of men who have sex with men (MSM), the prevalence of MA use among PLWH is approximately twice that observed in HIV-negative counterparts [2].

Behavioral and biomedical interactions between HIV and MA likely drive this overlap: MA use is associated with high-risk sexual behaviors that facilitate HIV transmission [3,4], and once infected, individuals who use MA often experience suboptimal HIV treatment outcomes [5]. PLWH who use stimulants exhibit lower adherence to antiretroviral therapy (ART) and achieve viral suppression more slowly, resulting in higher rates of detectable viremia [6]. A variety of co-morbid factors contribute to neurocognitive impairment in PLWH, including aging, cardiovascular disease, chronic inflammation, and substance use [7,8,9,10]. Within this context, MA use has emerged as an important and common co-morbidity that may exacerbate neurocognitive decline in PLWH [11].

Damage involves dopaminergic circuitry across the striatum, cortex, hippocampus, and basal ganglia, with downstream impairments in learning, memory, executive function, emotion regulation, and psychomotor performance [12]. Damage involves dopaminergic circuits across the striatum, cortex, hippocampus, and basal ganglia, with downstream impairments in learning, memory, executive function, emotion regulation, and psychomotor performance [13]. In parallel, PLWH remains susceptible to HIV-associated neurocognitive disorder (HAND), which now predominantly presents in milder forms despite widespread ART use [14]. HIV gains access to the central nervous system (CNS) early in infection via a “Trojan horse” mechanism, whereby infected monocytes infiltrate the CNS. Once established, long-lived macrophages and activated microglia maintain persistent neuroinflammation [15,16]. Viral proteins (e.g., gp120, Tat) further amplify oxidative stress, glutamatergic excitotoxicity, blood–brain barrier (BBB) dysfunction, and pro-inflammatory cytokine cascades [17].

Co-occurring MA use and HIV infection may therefore produce synergistic neurotoxicity. MA upregulates CCR5 expression on myeloid cells, increases BBB permeability through tight-junction disruption and induction of matrix metalloproteinase-9 (MMP-9), enhances monocyte transmigration in response to CCL2, and elevates pro-inflammatory cytokines (IL-1β, IL-6, TNF-α) [18]. Observational studies have reported higher rates of neuropsychological impairment, particularly in delayed recall and working memory—among MA-using PLWH, although findings across cognitive domains remain somewhat inconsistent [19]. Importantly, both HAND and MA-related cognitive deficits can compromise prospective memory and substantially increase the risk of ART nonadherence, thereby jeopardizing viral suppression and clinical outcomes [20,21,22,23].

Given these risks and the limited efficacy of current pharmacotherapies under investigation for MA use disorder, there is a critical need for objective, non-invasive biomarkers to aid diagnosis, risk stratification, treatment monitoring, and relapse prevention, especially in settings where lumbar puncture is impractical. A growing body of evidence supports blood-based markers of neuroinflammation and neuroglial injury, including sCD14 (monocyte activation), NSE and NfL (neuronal/axonal injury and BBB dysfunction), and S100B (astroglia injury) as promising biomarkers of neurocognitive impairment in MA users [24,25].

In this context, the main objective of the present study was to characterize peripheral blood biomarkers of immune activation/inflammation and BBB/neuronal injury in individuals with and without HIV, stratified by MA use, and to examine their relationships with cognitive performance. We hypothesized that MA-using PLWH would exhibit a distinct biomarker signature consistent with heightened neuroinflammation and BBB/neuronal injury, and that these profiles would correlate with domain-specific cognitive deficits. By focusing on accessible blood-based readouts in a high-burden setting, this work aims to inform clinically actionable tools for neuro HIV care and the management of MA use disorder.

## 2. Materials and Methods

### 2.1. Study Design and Ethical Approval

This cross-sectional study was conducted at the HIV Clinic of Hospital Civil de Guadalajara (Jalisco, Mexico). The protocol was approved by the Ethics Committee of Hospital Civil de Guadalajara (approval code CEI 208/23), and the study was performed in accordance with the principles of the World Medical Association Declaration of Helsinki. All participants provided written informed consent prior to screening and data collection.

### 2.2. Study Population and Clinical Evaluation

Participants were recruited between June 2023 and May 2025 and assigned to one of three study groups based on predefined inclusion criteria: (1) PLWH with active MA use, referred to as the MAHIV group; (2) PLWH with no history of MA use; and (3) HIV-negative Controls with no history of MA use.

Inclusion criteria were as follows: adults aged ≥ 18 years; PLWH were required to have been on ART for ≥1 year with suppressed HIV-1 viral load (<200 copies/mL). Participants in the MAHIV group had to be active MA users meeting the ICD-11 criteria for a harmful pattern of stimulant use involving MA (code 6C46.1, version January 2023) for at least 12 months if use was episodic, or at least 1 month if use was continuous (daily or almost daily). Healthy control subjects had no lifetime history of active illicit drug use.

Exclusion criteria were dependence on alcohol or any other drug (per DSM-5-TR criteria); untreated chronic hepatitis C infection; neurosyphilis; a history of CNS opportunistic infections, epilepsy, or stroke; advanced cognitive impairment precluding neuropsychological testing; inability to read or write; and a history of moderate or severe traumatic brain injury.

Non-MA users (PLWH and HIV-negative Controls) were matched to the MAHIV group by age and sex. All enrolled participants underwent a comprehensive medical history interview, including assessment of past and current use of tobacco, alcohol, and other substances, as well as comorbid medical conditions, and a complete physical examination. As part of the screening procedures, all participants received rapid blood tests for syphilis and hepatitis C to confirm the absence of these infections according to the exclusion criteria. Additionally, MA-using HIV-negative participants were given a rapid HIV test to verify their seronegative status.

A urine toxicology screen was performed for each participant using a qualitative immunochromatographic multi-drug panel (BIO-DRUG 6×1, cat. #2001107, MexLab, Zapopan, Mexico) to detect the presence of cocaine, opiates, cannabis (THC), benzodiazepines, amphetamines, and MA. The urine drug screen was conducted according to the manufacturer’s instructions, and the results were interpreted after 20 min.

### 2.3. Neuropsychological Assessment

All participants completed a battery of standardized neuropsychiatric and cognitive tests administered by trained personnel. Global cognition was evaluated with the Montreal Cognitive Assessment (MoCA). Substance use and addiction risk were screened using the Alcohol, Smoking, and Substance Involvement Screening Test (ASSIST). Affective and sleep-related symptoms were assessed with the Beck Depression Inventory-II (BDI-II), the Generalized Anxiety Disorder 7-item scale (GAD-7), and the Pittsburgh Sleep Quality Index (PSQI). These instruments provided quantitative measures of cognitive performance and neuropsychiatric symptoms for subsequent analyses.

### 2.4. Laboratory and Biomarker Analyses

All laboratory tests were performed on fasting blood samples. In total, 15 mL of peripheral blood was collected from each participant and distributed into three tubes for processing: 1. EDTA tube (6 mL): used for a complete blood count (CBC) and CD4^+^ T-cell count, measured by flow cytometry (AQUIOS R flow cytometer, Beckman Coulter, Miami, FL, USA). Plasma from this EDTA tube was also isolated to quantify HIV-1 RNA viral load by real-time reverse-transcription PCR (Abbott Alinity m HIV-1 assay, Abbott Laboratories, Abbott Park, IL, USA); 2. Serum tube (6 mL, no anticoagulant): used for routine biochemical analyses, including fasting glucose, insulin, glycated hemoglobin (HbA1c), renal function markers (creatinine, urea), lipid profile (total cholesterol, low-density lipoprotein [LDL], high-density lipoprotein [HDL], very-low-density lipoprotein [VLDL], triglycerides), liver function enzymes (aspartate aminotransferase [AST], alanine aminotransferase [ALT], gamma-glutamyl transferase [GGT], alkaline phosphatase), and total bilirubin. All parameters were measured in Central Laboratory of Civil Hospital of Guadalajara “Fray Antonio Alcalde “using photometric and potentiometric methods on an automated chemistry analyzer (AU5800 autoanalyzer, Beckman Coulter^®^, Brea, CA, USA); 3. Additional tube (3 mL): the remaining blood was processed and stored as plasma/serum for research assays of neuroinflammatory and neuronal injury biomarkers (described below).

Circulating biomarkers were quantified by enzyme-linked immunosorbent assay (ELISA) using commercial kits: Human soluble CD14 (sCD14) ELISA kit (catalog ID: DC140, R&D Systems, Minneapolis, MN, USA); Human neuron-specific enolase (NSE) ELISA kit, Abclonal (catalog ID: RK01966-96T, ABclonal Science, Wuhan, China); Human S100 calcium-binding protein B (S100B) ELISA kit, (catalog ID: EEL045, Invitrogen, Carlsbad, CA, USA)); and Human neurofilament light chain (NEFL) ELISA kit (catalog ID: E-EL-H26203, Elabscience, Houston, TX, USA). All ELISA assays were performed strictly according to the manufacturer’s instructions. Absorbance for each ELISA plate was measured at the specified wavelength for the target analyte using a Biotek Synergy H1 microplate reader (Agilent Technologies^®^, Santa Clara, CA, USA).

### 2.5. Statistical Analysis

The sample size was calculated a priori based on the findings of Zhu et al. [25], who reported a significant difference in NSE levels among MA users. The formula used to estimate the sample size was determined for estimating population means (s = 0.01, d = 0.02) and indicated a total number of 117 participants (approximately 39 per group). With this size, the study has a statistical power of 80% to detect differences of this magnitude with a bilateral significance level of 5%. Cohen’s effect corresponds to 0.18.

Normality was first assessed to determine data distribution using the Shapiro–Wilk test. Comparisons between two independent groups were conducted using the Mann–Whitney *U* test. Comparisons among more than two independent groups were conducted using Kruskal–Wallis’s test for non-normally distributed data, post-hoc pairwise comparisons following the Kruskal–Wallis’s test were performed using Dunn’s test with Bonferroni correction for multiple comparisons. Associations between variables were assessed exclusively using Spearman’s rank correlation coefficient due to the non-normal distribution of the variables of interest; therefore, Pearson correlation analyses were not applied. For the analysis of proportions (categorical variables), the chi-square test was used when expected frequencies were adequate (expected cell count ≥ 5), whereas Fisher’s exact test was applied when expected frequencies were low (expected cell count < 5). Normally distributed data are presented as mean ± standard deviation, whereas non-normally distributed data are reported as median and interquartile range (IQR). A two-sided *p*-value < 0.05 was considered statistically significant. Finally, multivariable linear regression analyses were applied only to continuous outcomes. The use of linear models was justified by the objective of estimating adjusted associations between group status and outcome variables while accounting for potential confounding factors. Covariates (age, education, and sexual orientation) were selected a priori based on biological plausibility and previous literature. Model assumptions were assessed using standard diagnostic procedures, including evaluation of linearity, normality of residuals, homoscedasticity, and multicollinearity, prior to interpretation of the regression results. Adjusted regression coefficients (β) were reported to estimate the independent association between group status and each outcome. This approach was used to independently adjust the results and to assess the robustness and validity of the observed associations after accounting for potential confounding factors. In addition, the effect size of the sCD14 biomarker was estimated using Cliff’s delta with 95% confidence intervals, providing a non-parametric measure of the magnitude of the difference between groups that is independent of data distribution assumptions. All statistical analyses were performed using RStudio version 2025.09.2+418 and GraphPad Prism [computer software] version 10.6.1, San Diego, CA, USA.

## 3. Results

### 3.1. Participants

We analyzed 121 participants: the MAHIV group (PLWH with MA use; *n* = 40), the PLWH group without MA use (*n* = 42), and the HIV-negative Control group (*n* = 39). Each group included one woman to preserve sex matching. The MAHIV and PLWH groups had similar age distributions, whereas the Control group was significantly younger. The MAHIV group had a lower BMI compared with PLWH but also reported the lowest levels of physical activity (MAHIV vs. PLWH, *p* = *0.006*; MAHIV vs. Control, *p* = *0.0002*). Sociodemographic characteristics are summarized in Table 1.

### 3.2. Antiretroviral Therapy (ART) Discontinuation

All PLWH, with and without MA use, were in virologic control at the time of evaluation. Participants had similar durations of HIV infection and ART exposure, as well as comparable CD4^+^ T-cell counts. Most individuals were classified as immune responders (CD4^+^ T-cell count > 200 cells/μL), whereas 10% of the MAHIV group were non-immune responders (CD4^+^ T-cell count < 200 cells/μL). Importantly, the MAHIV group had a significantly higher rate of ART discontinuation (62.5%) than PLWH without MA use (38.0%; *p < 0.0001*; Table 2). However, at the time of study enrollment, all participants were adherent to ART, as evidenced by an undetectable HIV viral load, consistent with the study’s inclusion criteria. Nonadherence and treatment interruption are clinically relevant because they can facilitate the emergence of drug resistance and accelerate HIV disease progression.

### 3.3. Substance Use

In the MAHIV group, the main substances currently used were MA and crystal meth, and concurrent use of two or more drugs was frequent (Table 3). The primary route of MA administration was smoking (57.5%), followed by snorting (15.0%), injection (17.5%), and combined routes (10.0%). The median time since last substance use was 7 days (IQR: 1.75–60), and 40.7% reported daily MA use, 28.5% weekly use, 12.0% monthly use, and 2.3% sporadic use within the preceding year, in accordance with the inclusion criteria. The mean age at onset of MA use was 29.2 ± 9.0 years.

Regarding lifetime substance use history in the MAHIV group, the age of onset of drug use was 29.3 ± 9.1 years. All participants (100%) reported having consumed alcohol at least once, 97.5% had used tobacco, 90.0% cannabis (11.0% ingested, 89.0% smoked), 77.5% cocaine, and 60.0% inhalants (58.3% poppers, 4.1% gasoline, 8.3% glue, and 25.0% other substances). In addition, 47.5% had used tranquilizers, 32.5% hallucinogens (30.8% LSD, 30.8% ketamine, 7.7% “acids”, 7.7% PCP, and 23.0% other substances), 5.0% opioids (heroin), and 11 participants reported having used drugs associated with ancestral rituals (15.0% peyote and 12.5% ayahuasca).

In the PLWH group without MA use, 83.3% used tobacco at least once in their lifetime, 100% had consumed alcohol, 69.0% had used marijuana (76.0% smoked, 24.0% ingested in food), 26.0% had tried cocaine, and 35.5% had used inhalation at least once (84.0% poppers, 4.0% gasoline, 12.0% other substances). Additionally, 12.0% reported tranquilizer use, 4.8% hallucinogen use (PCP), 4.8% opioid use (heroin), and 7.0% other substances (mainly ayahuasca).

In the Control group, 100% had tried alcohol, 83.0% tobacco, 68.3% marijuana (19.5% ingested and 80.5% smoked), 12.2% cocaine, 7.3% inhalants (poppers), 12.2% tranquilizers, 12.2% hallucinogens (LSD), and 17.0% reported having used other unspecified substances.

In both the PLWH and Control group, two participants reported lifetime MA use; however, this occurred outside the time window specified in the inclusion criteria and was therefore not considered active use. This information was corroborated by negative urinary drug screening results for MA and related substances in those participants.

### 3.4. Substance Use During Sexual Activity (Chemsex) and Risk Behaviors

MA use during sexual activity (chemsex) was common in the MAHIV group: 27.0% reported MA use alone, 30.0% MA plus one additional drug, and 43.0%, MA plus two or more drugs (Table 4). MA use and polydrug consumption in sexual contexts were associated with increased sexual desire, disinhibition, and prolonged sexual encounters, typically linked to higher tolerance and dependence, complex withdrawal syndromes, and increased psychiatric morbidity.

Compared with the other groups, MAHIV participants displayed a higher frequency of condomless sex, a greater number of sexual partners, more transactional sex (sex in exchange for drugs), and a higher incidence of sexually transmitted infections (Table 4). These behaviors frequently involved polydrug use and drug consumption during ART, thereby increasing the risk of treatment discontinuation (Table 2). In the MAHIV group, such high-risk behaviors could occur in isolation (3.0%), as multiple concurrent risk behaviors (3.0%), or in combination patterns (25.0%).

### 3.5. Psychological and Cognitive Characteristics of Methamphetamine Users: Depression, Anxiety, and Sleep Quality

Global cognitive performance, assessed by the MoCA, was significantly lower in both MAHIV and PLWH groups compared with Controls (*p* < 0.001 and *p* = 0.0145, respectively), with the greatest deficit observed in MAHIV (median score 22, IQR: 18–25; Figure 1A).

In the MAHIV group, domain-level MoCA analysis showed significant impairments in executive/visuospatial functions, memory (delayed recall), attention, and language, indicating a detrimental impact of MA use on multiple clinically relevant cognitive domains. The most affected domain was memory/delayed recall (*p < 0.0001*), followed by executive/visuospatial functions (*p = 0.01*) and, to a lesser extent, language (*p = 0.0508*) (Table 5). Overall, 50.0% of MAHIV participants met criteria for mild cognitive impairment and 32.5% for cognitive impairment, whereas only 10.0% had no cognitive impairment (Table 5).

**Figure 1:**
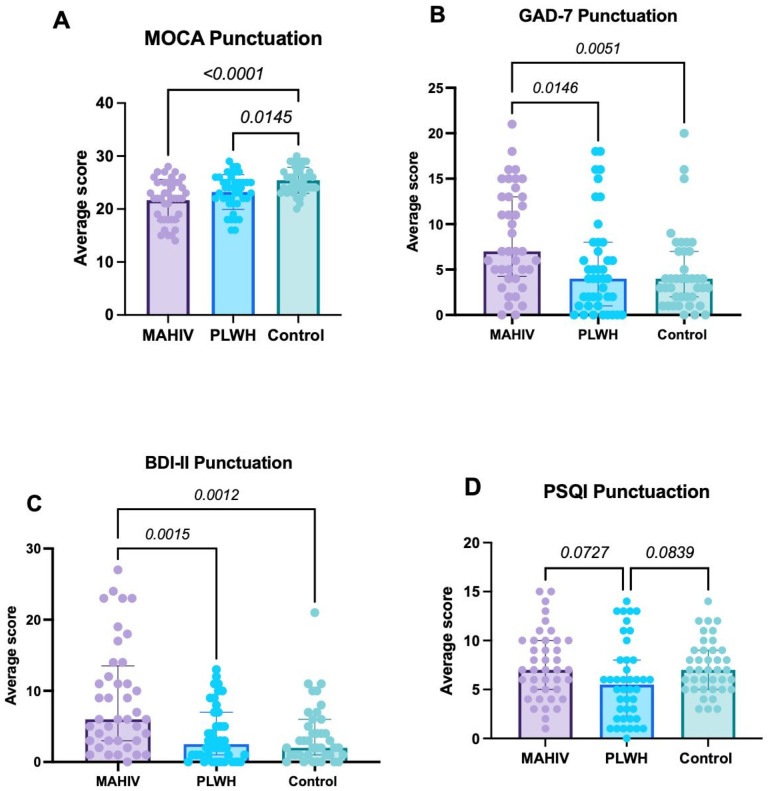
Scores derived from the use of instruments designed to assess Psychological and Cognitive Characteristics. (**A**) Score derived from the MoCA test; (**B**) Presents the scores obtained from the GAD-7 (anxiety); (**C**) Displays the results from the BDI-II (depression); and (**D**) Shows the results obtained from the PSQI (sleep quality). Values represented in the graphs show the median and interquartile ranges (IQR 1–3). Data were analyzed using Kruskal–Wallis’s test with Dunn’s post hoc test; *p*-value < 0.05 was considered statistically significant. MAHIV: Methamphetamine users who live with HIV, PLWH: People that lives with HIV, HIV: Human Immunodeficiency virus, MOCA: Montreal Cognitive Assessment, GAD-7: Generalized Anxiety Disorder BDI-II: Beck’s depression inventory, PSQI: Pittsburgh Sleep Quality Index.

Regarding anxiety, GAD-7 scores were significantly higher in the MAHIV group (median 7, IQR: 4.25–13) than in the other two groups (overall *p* = 0.026). The largest difference was observed between MAHIV and Controls (median 4, IQR: 2–7; *p* = 0.0051), and the difference between MAHIV and PLWH (median 4, IQR: 1–8) was also significant (*p* = 0.0146). No significant difference in GAD-7 scores was found between PLWH and Controls (Figure 1B). These results indicate a substantially higher anxiety burden among PLWH who use MA; notably, 42.5% of MAHIV participants met criteria for anxiety.

With respect to depressive symptoms, MAHIV participants had significantly higher BDI-II scores (median 6, IQR: 3–13.5) than both Controls (median 2, IQR: 1–6; *p* = 0.0012) and PLWH (median 2.5, IQR: 1–7; *p* = 0.0015), with no difference between Controls and PLWH (Figure 1C). Importantly, 70.0% of MAHIV participants obtained BDI-II scores consistent with mild to moderate depression (Table 6). These data were consistent with the proportion of participants who already had a formal psychiatric diagnosis, most commonly depression, anxiety, or both.

In the PSQI-based assessment of sleep quality, no statistically significant differences were observed in total PSQI score among groups (MAHIV: median 7, IQR: 5–10; Controls: median 7, IQR: 5–9; PLWH: median 5, IQR: 2–8). However, the MAHIV group showed the highest prevalence of clinically relevant sleep disturbances, followed by PLWH and Controls (47.5% vs. 21.4% vs. 20.5%, respectively; Figure 1D). Component-wise analysis revealed pronounced disturbances in the MAHIV group, with shorter sleep duration, greater sleep latency, reduced sleep efficiency, and more prominent daytime dysfunction (Table 7). These component-level abnormalities are consistent with potential negative effects on memory, attention, learning, and decision-making.

It is noteworthy that the Control group also presented relatively elevated PSQI scores, particularly in sleep quality, sleep duration, and daytime functioning. This pattern likely reflects the characteristics of the Control group, composed of working-age, economically active adults, most of whom were employed in service-related occupations requiring high professional and emotional demands and often long or irregular working hours.

### 3.6. Blood Biomarkers Related to Inflammation and Neuronal Damage

#### 3.6.1. Soluble CD14 (sCD14)

**Figure 2:**
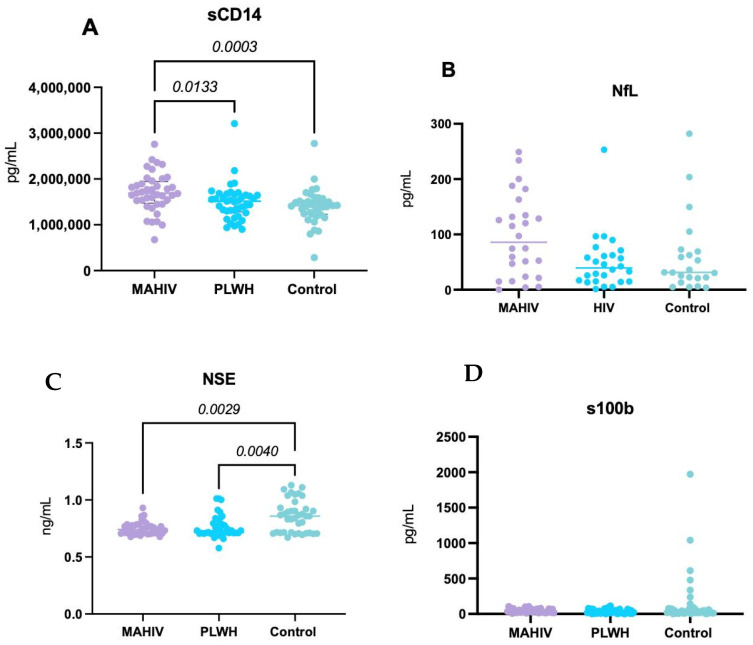
Systemic concentrations of biomarkers related to Inflammation and Neuronal Damage. (**A**) sCD14 concentrations; (**B**) NfL concentrations; (**C**) NSE concentrations and (**D**) S100b. All the biomarkers were measured in serum samples (blood-derived) with commercially available ELISA kits. Values represented in the graphs show the median and interquartile ranges (IQR 1–3). Kruskal–Wallis’s test with Dunn’s post hoc test *p*-value < 0.05 was considered statistically significant. MAHIV: Methamphetamine users who live with HIV, PLWH: People Living with HIV, HIV: Human Immunodeficiency virus, sCD14: soluble CD14, NfL: Neurofilament light chain, NSE: Neuron-specific enolase, S100b: S100 calcium-binding protein B.

Serum sCD14 concentrations were significantly higher in the MAHIV group than in PLWH without MA use and Controls (*p* < 0.01 and *p* < 0.0001, respectively), consistent with heightened monocyte activation and systemic inflammation in MA users, beyond the effects attributable to HIV infection alone (Figure 2A).

#### 3.6.2. Neurofilament Light Chain (NfL)

No statistically significant differences in serum NfL concentrations were observed among the three groups. However, there was a trend toward higher values in MAHIV compared with both PLWH and Controls (Figure 2B), suggesting possible subclinical axonal injury that may not have reached statistical significance in this sample.

#### 3.6.3. Neuron-Specific Enolase (NSE)

Both the MAHIV and PLWH groups exhibited significantly higher serum NSE concentrations—a marker of neuronal injury—than Controls (*p* < 0.0001 and *p* < 0.01, respectively), with the largest difference observed between MAHIV and Controls (Figure 2C). These findings suggest greater neuronal damage in PLWH, particularly in those with concurrent MA use.

#### 3.6.4. S100B Concentrations

No significant differences were found in serum S100B concentrations among the three groups, although there was a trend toward higher S100B levels in MAHIV compared with PLWH and Controls (Figure 2D).

### 3.7. Biomarkers Correlations with Global Cognition, Depression, and Anxiety

**Figure 3:**
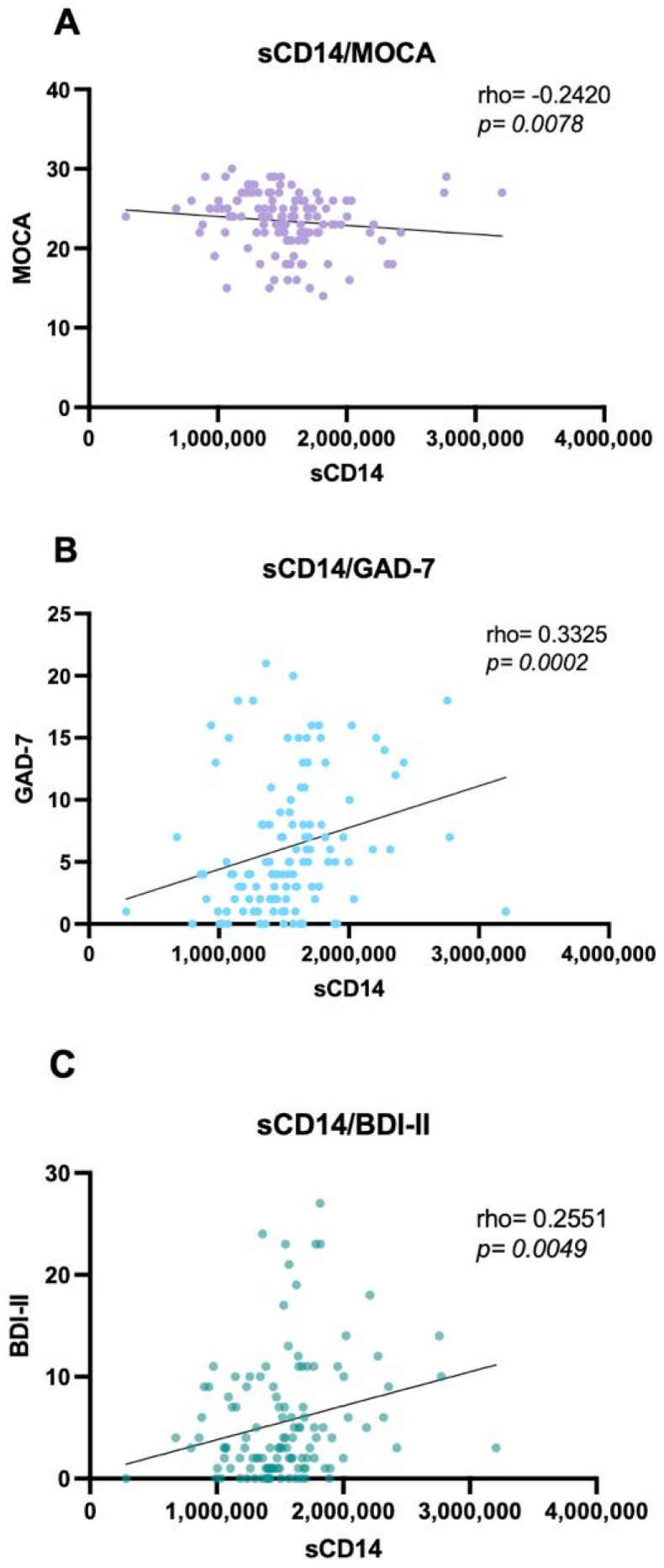
sCD14 correlations with global cognition, depression, and anxiety. (**A**) Negative correlation between sCD14 concentrations and the MoCA test scores (rho = −0.24; *p = 0.005*); (**B**) Correlation between sCD14 concentrations and GAD-7 (rho= 0.33; *p = 0.0005*); (**C**) Correlation between sCD14 concentrations and BDI-II (rho = 0.25; *p = 0.005*); Data were analyzed using the Correlation Spearman test; *p*-value < 0.05 was considered statistically significant. MAHIV: Methamphetamine users who live with HIV, PLWH: People Living with HIV, HIV: Human Immunodeficiency virus, MOCA: Montreal Cognitive Assessment, GAD-7: Generalized Anxiety Disorder BDI-II: Beck’s depression inventory.

A significant negative correlation was observed between global cognitive performance (MoCA) and sCD14 concentrations (rho = −0.24; *p* = 0.005), indicating that higher immune activation/inflammation is associated with poorer cognition (Figure 3, Table 8). Consistent with its role as a marker of chronic monocyte-driven immune activation, elevated sCD14 was associated with lower MoCA scores, supporting immune activation as a candidate mechanism for cognitive impairment.

Additionally, sCD14 showed modest but significant positive correlations with both anxiety and depression scores (GAD-7 and BDI-II), indicating that higher levels of inflammation—reflected by increased sCD14—are associated with greater depressive and anxiety symptom burden (Figure 3, Table 8). No other biomarkers (NfL, NSE, and S100B) showed significant correlations with psychological or cognitive instruments.

### 3.8. Group Differences After Adjustment for Demographic Variables (Age, Education, and Sexual Orientation) and Effect Size Assessment of sCD14

To account for demographic differences across study groups, multivariable linear regression analyses were conducted for all cognitive, psychological, and biomarker outcomes. Group status was specified as the primary independent variable, while age, education, and sexual orientation were included as covariates. Adjusted regression coefficients (β) were reported to estimate the independent association between group status and each outcome (Table 9).

After adjustment, significant group differences persisted for MoCA scores, anxiety, and depressive symptom measures (GAD-7 and BDI-II), as well as for inflammatory and neuronal biomarkers (sCD14 and NSE). These findings indicate that the observed associations were independent of demographic differences between groups.

In addition to the multivariable regression analyses, effect size estimation using Cliff’s delta was performed exclusively for sCD14. A moderate effect size was observed between groups (Cliff’s Δ = 0.40, 95% CI 0.15–0.59), which indicates a consistent and meaningful difference between groups, supporting its potential relevance beyond statistical significance.

### 3.9. Brief Clinical–Neuropsychological Interpretation

The MAHIV group was characterized by higher rates of ART discontinuation, lower physical activity, and multiple high-risk behaviors—including marked polydrug use and chemsex—patterns that are typically associated with disease progression, poorer mental health, and an elevated risk of sexually transmitted infections. Emotionally, MAHIV participants exhibited a heavier burden of depressive and anxiety symptoms and shorter sleep duration. Cognitively, they showed worse global performance and deficits across key domains (memory, attention, language, and abstraction).

At the biological level, serum sCD14 (a marker of immune activation/inflammation) and NSE (a marker of neuronal injury) were significantly elevated in MAHIV. Notably, higher sCD14 concentrations correlated with lower MoCA scores, implicating chronic immune activation as a potential mechanism underlying the observed cognitive impairment. Taking together, these clinical, neuropsychological, and biomarker findings support the hypothesis that MA use in PLWH compounds neuropsychological vulnerability, with elevated NSE and sCD14 providing convergent evidence of neuronal injury and systemic immune activation. Such biomarkers may help elucidate the neurobiological pathways of MA use and could ultimately inform risk stratification, prognostication, and the development of targeted therapeutic strategies in this high-risk population.

## 4. Discussion

In this cross-sectional study of 121 adults (40 PLWH with active MA use, 42 PLWH without MA use, and 39 HIV-negative Controls), we observed a convergent pattern of biomedical, neurocognitive, and psychosocial vulnerability in the MA-using PLWH group (MAHIV). MAHIV participants had higher rates of ART discontinuation, lower physical activity, frequent high risk sexualized polysubstance use (chemsex), a heavier emotional burden (depression and anxiety), shorter sleep, poorer global cognition with prominent deficits in executive function, memory, attention, and language, and elevated circulating NSE and sCD14. Notably, higher sCD14 concentrations correlated inversely with global cognition (MoCA) and positively with anxiety (GAD-7) and depression (BDI-II), implicating chronic monocyte-driven immune activation in both cognitive compromise and psychiatric symptomatology. Taken together, these findings support the view that MA use acts as both a behavioral and biological amplifier of neurocognitive vulnerability in PLWH.

We found a higher rate of ART interruption and lower physical activity levels among MAHIV participants. These observations are consistent with studies in MSM and other HIV-affected populations, where recent MA use is independently associated with non-adherence and worse virological outcomes [5,6]. Chemsex involving MA, mephedrone, and cocaine has been linked to more sexual partners, reduced condom use, higher-risk practices, and increased STI incidence [26,27,28,29]. Polysubstance use was frequently observed in the MAHIV group, consistent with population-level and clinical evidence showing high rates of co-occurring substance use among people who use MA [30]. However, all participants in our study met the ICD-11 criteria for a harmful pattern of stimulant use involving MA. Thus, our polysubstance-use and sexual-risk data align with contemporary literature and reinforce the need for integrated harm-reduction and combination-prevention strategies.

MAHIV participants reported a markedly higher burden of depressive symptoms and generalized anxiety than both HIV-negative Controls and PLWH without MA use. In PLWH, the bidirectional interplay between mental health and clinical outcomes is well recognized: depression and anxiety impair adherence and self-regulation, while stimulant use exacerbates affective symptoms [31,32,33]. MAHIV individuals also reported shorter sleep duration, a clinically relevant feature because chronic sleep deficiency in MSM—including MSM living with HIV—has been associated with more frequent condomless anal sex, a greater number of sexual partners, and missed ART doses [34]. Sleep restriction and circadian disruption impair attention, working memory, and executive control, and can potentiate impulsive or reward-driven decision-making, especially in stimulant-using populations [35,36,37].

These findings suggest a reinforcing loop: MA use increases psychiatric distress and sleep dysregulation; in turn, mood symptoms and sleep loss worsen ART adherence and sexual risk-taking, thereby intensifying both HIV-related and psychosocial harms [34,38]. The convergence of high depressive/anxiety burden, shortened sleep, and high-risk sexualized polysubstance use that we observed in MAHIV points to an especially fragile neurobehavioral profile that is unlikely to respond adequately to siloed interventions. Instead, it may require integrated care models that simultaneously address mental health, sleep hygiene, addiction, and HIV management [39,40].

Neurocognitive performance was significantly worse in the MAHIV group. Both PLWH groups scored lower than HIV-negative Controls on global cognition (MoCA), with the largest decrement observed in MAHIV. Of note, cognitive performance was evaluated using a brief screening instrument rather than a comprehensive neuropsychological battery, which may limit the granularity of domain-specific inferences. Within MAHIV, domain-level analyses nonetheless suggested deficits in executive function, memory, attention, language, and (at the trend level) abstraction, whereas orientation appeared relatively preserved. This cognitive profile—frontally mediated executive/attention deficits coupled with episodic memory compromise—is consistent with HIV-associated neurocognitive disorder (HAND), which remains prevalent in approximately 40–50% of PLWH even in the contemporary ART era and in the absence of major confounding neurological disease [9].

MA use may magnify these HAND-like deficits. Chronic MA exposure disrupts dopaminergic and front striatal circuits, impairs inhibitory control, heightens impulsivity, and degrades decision-making and working memory; these liabilities are repeatedly linked to risk-taking sexual behavior and difficulty sustaining goal-directed health behaviors [41]. Importantly, cognitive and behavioral dysregulation can persist even after recent abstinence from MA, including impairments in problem solving, language-based reasoning, reward/risk evaluation, and inhibitory control compared with drug-free adults [42,43,44]. The MAHIV cognitive pattern we observed—broad deficits across executive function, attention, and memory—aligns with this literature and suggests an additive or synergistic neurotoxic effect of MA on top of chronic HIV-related neuroinflammation [19,45]. Clinically, these findings imply that active MA use in PLWH may accelerate functional cognitive aging and interfere with medication management, safer-sex negotiation, and daily self-care.

Beyond behavior and cognition, our data reveals a biological signature. Circulating sCD14—an established marker of monocyte activation and systemic inflammation—was significantly higher in MAHIV than in both PLWH without MA use and HIV-negative Controls. NSE, a glycolytic enzyme predominantly expressed by neurons and released into the circulation with neuronal injury or (BBB disruption [46], was also higher in MAHIV (and, to a lesser extent, in PLWH without MA) than in Controls.

In addition to the direct neurotoxic effects of HIV and MA, sleep disruption may contribute to the elevated NSE concentrations observed in our cohort. The MAHIV group showed the highest prevalence of poor sleep quality, with shorter sleep duration, longer sleep latency, reduced habitual sleep efficiency, and more pronounced daytime dysfunction compared with PLWH without MA use and HIV-negative control features consistent with clinically relevant sleep fragmentation and restriction. Experimental data in healthy men demonstrate that even a single night of total sleep deprivation can increase morning serum NSE and S100B levels by approximately 20%, suggesting that acute sleep loss induces neuronal stress, BBB perturbation, or both [47]. Taking together, our findings raise the possibility that chronic or recurrent sleep disturbance—superimposed on HIV infection and MA exposure—may further amplify neuronal injury signals captured by blood-based biomarkers such as NSE in MAHIV individuals.

By contrast, group differences in NfL and S100B were less pronounced, although both showed a tendency toward higher levels in MAHIV. These results fit with emerging evidence that MA and HIV each drive chronic immune activation, endothelial dysfunction, and neurovascular stress—mechanisms linked to cerebrovascular and neurocognitive morbidity [19,48,49]. Importantly, all biomarkers in our study were measured in blood, suggesting that they may represent non-invasive neuroinflammation monitoring strategies that complement conventional neuroimaging.

MA exposure has been associated with upregulation of inflammatory chemokines (e.g., CCL2/MCP-1), adhesion molecules (ICAM-1, VCAM-1), and cytokines that facilitate monocyte trafficking across the BBB, while HIV itself sustains a pro-inflammatory milieu despite effective ART [50]. Elevated non-classical/activated monocyte phenotypes and soluble monocyte activation markers such as sCD14 have been repeatedly implicated in cognitive impairment among PLWH, including in cohorts with cerebral small-vessel disease and otherwise suppressed viremia [51,52,53].

Independent studies in virally suppressed PLWH have shown that higher plasma sCD14 and related chemokines (e.g., MCP-1) are associated with worse neurocognitive performance, supporting a model in which persistent monocyte-driven inflammation contributes to HAND pathogenesis even in the ART era [52,54].

The significant inverse correlation between sCD14 and global cognition (MoCA) in our sample (ρ ≈ −0.24; *p* = 0.005) provides direct clinical evidence that chronic peripheral immune activation tracks with cognitive dysfunction in MA-using PLWH. In parallel, NSE functions as a peripheral biomarker of neuronal injury; reviews and studies in MA dependence show that NSE increases with neuronal damage and that non-pharmacological interventions such as aerobic exercise can reduce NSE and S100B, suggesting neuroprotective potential or partial reversibility [25,55,56]. Altogether, our biomarker findings argue that MA use in PLWH accentuates a chronic inflammatory state (indexed by sCD14) and is accompanied by biochemical evidence of neuronal injury (NSE).

Preclinical and clinical data show that MA increases BBB permeability and disrupts tight-junction protein expression, while chronic CNS inflammation persists in HIV despite ART [48,56,57,58]. This framework is consistent with the elevated sCD14 (systemic monocyte activation) and NSE (neuronal injury) observed in our study, as well as with the pattern of executive and attentional deficits.

Within the MAHIV group, higher sCD14 levels were also associated with greater depressive symptom burden (BDI-II) and higher generalized anxiety scores (GAD-7), reinforcing an inflammatory–affective phenotype in MA-using PLWH. This pattern aligns with prior evidence in PLWH showing that affective symptomatology tracks innate immune activation: in the Veterans Aging Cohort Study, somatic depressive symptoms remained independently associated with elevated circulating sCD14 after multivariable adjustment [59]. Soluble biomarkers reflecting monocyte/macrophage activation (including sCD14 and sCD163) have been linked to both cognitive outcomes and depressive symptoms in PLWH in the combination-therapy era [60]. In HIV-negative populations, longitudinal data show that endotoxin-related immune activation—reflected by higher LBP/sCD14—predicts subsequent depressive symptoms [61]. This is consistent with a recent meta-analysis demonstrating that biomarkers of gut permeability and inflammation (intestinal fatty-acid binding protein [I-FABP], zonulin, antibodies to endotoxins, and sCD14) are associated with depressive symptoms, reinforcing the notion that dysfunction of the gut–brain axis contributes to mental disorders, particularly depressive disorders [62].

The relationship between inflammation and depression remains a key research priority. A recent scoping and mechanistic systematic review propose a biomarker-driven framework of neuroimmunometabolic mechanisms that may underlie the increased risk of depression in people with HIV; chronically activated microglia—driving neuroinflammatory cascades upregulated in HIV—are posited as a central link between CNS infection and depressive symptomatology [63]. In complementary work, PLWH were more likely to report depressive symptoms than HIV-negative individuals, and biomarkers of central and peripheral inflammation partially mediated the association between HIV status and depressive symptoms [64]. Consistent with our findings, stimulant exposure has also been associated with heightened innate immune activation; in sexual minority men with and without HIV, MA and other stimulant use were linked to immune dysregulation, as evidenced by elevated monocyte-activation biomarkers such as sCD14 [65]. Similarly, a recent mechanistic review in HIV reports that MA use is associated with higher levels of inflammatory markers (hs-CRP, IL-6, sTNFR-1) and myeloid activation markers (sCD163, sCD14), with comparable elevations observed among PLWH who use other stimulants [66].

Evidence specifically linking sCD14 to anxiety is more limited but emerging: a pilot study in patients with moderate anxiety reported that symptomatic improvement following curcumin supplementation was accompanied by reductions in circulating sCD14 [67], and broader inflammatory models have associated immune activation with anxiety dimensions in large population cohorts [68].

Taken together, these findings are consistent with a gut–monocyte–brain immune-activation pathway, in which MA-related immune dysregulation amplifies affective burden in PLWH. This framework provides biological plausibility for the positive associations we observed between sCD14 and both depressive and anxiety symptoms in the MAHIV group and underscores the importance of targeting innate immune activation in parallel with standard psychiatric and substance-use interventions.

These results have several practical implications. First, PLWH who use MA represent a syndemic phenotype: addiction, mental health comorbidity, sleep disturbance, high-risk sexual networks, suboptimal ART adherence/suppression, and biologically measurable neuroinflammatory stress all cluster in the same individuals. Our findings support integrated interventions that simultaneously address stimulant use, mental health, sleep, cognition, and adherence. The most consistently supported strategy for stimulant use disorder is contingency management, complemented by harm reduction and integrated HIV-care models [69,70,71]. Intervention packages should include routine screening for MA and other substances; adherence supports (reminders, digital monitoring, pharmacy-assisted strategies); psychological treatment (cognitive-behavioral therapy, motivational interviewing) [72,73,74,75,76]; and, when feasible, structured exercise programs, given their potential neurobiological modulation (reductions in NSE/S100B) [25]. From a public health perspective, surveillance of chemsex and STI prevention (PrEP/PEP, vaccinations, safer-use materials, education on substance mixing) are priorities [27,28,29,77,78].

Second, our findings raise the possibility that peripheral inflammatory and neuronal injury markers could complement brief cognitive screening to identify high-risk patients earlier. sCD14 may help identify PLWH who use MA and are at heightened risk of neurocognitive impairment, potentially prior to the onset of overt functional disability. Third, these results underscore that neuroinflammation is not merely an epiphenomenon but a putative therapeutic target. Anti-inflammatory and neuroprotective strategies are being actively explored. For example, human data suggest that cannabis exposure may attenuate specific pro-inflammatory and endothelial activation markers (e.g., ICAM-1, VCAM-1, CCL2/MCP-1) in the context of MA use disorder and/or HIV, pointing toward cannabinoid pathways as potential adjunctive modulators of chronic inflammation [50]. Separately, structured aerobic exercise in MA-dependent individuals has been shown to improve cognitive performance and reduce circulating markers of neuronal injury such as NSE and S100B, potentially by repairing BBB integrity and mitigating neurotoxicity [25]. Although the effects of exercise and cannabinoid-based modulation were not tested in our cohort, these approaches illustrate plausible, non–ART-based levers to dampen neuroinflammation and protect cognition in high-risk PLWH.

Several limitations must be acknowledged. First, the present analysis is cross-sectional; causality cannot be inferred. Second, selection and information biases may affect self-reported substance use and sexual behaviors. Third, cognition was assessed using screening and domain-level measures rather than a full neuropsychological battery, and neuroimaging or CSF assays were not included. This represents an experimental limitation, as relying solely on serum biomarkers may reduce the sensitivity and specificity of observed associations and may not fully capture central nervous system changes. Consequently, although correlations were observed with neurocognitive performance (MoCA), depression, anxiety, and sleep quality, these results should be interpreted with caution, as they do not allow direct inference regarding the extent of CNS neuronal injury. Fourth, polysubstance use represents a potential confounder that may have affected both cognitive outcomes and biomarker alterations. Finally, we did not measure additional biomarkers (e.g., CCL2, GFAP, sTREM2) that could enrich mechanistic inference, and generalizability is limited beyond predominantly male participants. Strengths include multidimensional assessment (clinical/behavioral, mental health, sleep, cognition) with incorporation of blood biomarkers, explicit between-group comparisons (PLWH ± MA, HIV-negative Controls), and domain-specific cognitive analyses. Nonetheless, convergence across clinical, cognitive, and serologic findings strengthens the conclusion that MA uses functions as an aggravating factor for neuropsychological vulnerability in PLWH.

Longitudinal studies are needed to (i) delineate trajectories of cognitive and biomarker change with modification of MA use; (ii) evaluate the prognostic and treatment-response utility of sCD14 and NSE (alone and in combination with NfL/sGFAP); (iii) compare integrated intervention packages (contingency management ± CBT ± supervised exercise) on cognitive and adherence outcomes; and (iv) interrogate, via multimodal neuroimaging, relationships among peripheral immune activation (sCD14/sCD163), BBB permeability, and white-matter microstructure in MAHIV.

## 5. Conclusions

Among PLWH who use MA, we document a high-risk profile characterized by greater ART discontinuation, lower physical activity, and chemsex-related polysubstance use and behaviors associated with STIs. The MAHIV group showed a heavier emotional burden (depression and anxiety) and shorter sleep, factors linked to risk behaviors, poorer adherence, and worse cognitive performance. Global cognitive performance was poorer in MAHIV, with prominent deficits in executive function, memory, language, and attention. Elevated circulating markers of systemic immune activation (sCD14) and neuronal injury (NSE), together with the inverse association between sCD14 and MoCA scores, support chronic immune activation as a central pathway underlying cognitive impairment. Overall, our results support MA, uses as an aggravating factor for neuropsychological vulnerability in PLWH. Biomarkers such as sCD14 and NSE emerge as candidate tools with potential prognostic and monitoring value and as guides for designing targeted interventions in this high-risk population.

## Figures and Tables

**Table 1:** Sociodemographic characteristics.

	MAHIV	PLWH	Control	*p*
Females, *n* (%)	1 (2.5%)	1 (2.3%)	1 (2.5%)	*1 ^b^*
Males, *n* (%)	39 (97.5%)	41 (97.6%)	38 (97.4%)	
Age (years) ^median, IQR^	38 (33, 44.25)	38 (32.2, 43)	30 (25.5, 34)	*<0.0001 ^a^*
BMI (kg/m^2^) ^median, IQR^	23.10 (21.30, 24.47)	27.0 (24.81, 29.01)	24.86 (23.84, 27.02)	*<0.0001 ^a^*
**Identity**				
Men, *n* (%)	37 (92.5%)	41 (97.6%)	38 (97.4%)	*0.5277 ^b^*
Women, *n* (%)	3 (7.5%)	1 (2.3%)	1 (2.5%)	
Nonbinary, *n* (%)	0 (0%)	0 (0%)	0 (0%)	
**Orientation**				
Homosexual, *n* (%)	31 (77.5%)	37 (88.0%)	6 (15.3%)	*<0.0001 ^b^*
Heterosexual, *n* (%)	9 (22.5%)	5 (12.0%)	33 (84.6%)	
**Civil status**				
Single, *n* (%)	34 (85%)	25 (59.5%)	27 (69.2%)	*0.0015 ^b^*
Married, *n* (%)	0 (0%)	3 (7.1%)	8 (20.5%)	
Common-law marriage, *n* (%)	4 (10%)	13 (31%)	4 (10.2%)	
Divorced, *n* (%)	1 (2.5%)	0 (0%)	0 (0%)	
Widower, *n* (%)	1 (2.5%)	1 (2.4%)	0 (0%)	
Study time (years) ^median, IQR^	12 (9, 14)	13 (12, 16)	19 (17, 22)	*<0.0001 ^a^*
Children, *n* (%)	11 (27.5%)	5 (12%)	6 (15.3%)	*0.1779 ^b^*
Physical Activity, *n* (%)	10 (25%)	23 (54.7%)	27 (69.2%)	*0.0002 ^b^*
Comorbidities, *n* (%)	10 (25%)	11 (26%)	13 (33.3%)	*0.6955 ^b^*

MAHIV: Methamphetamine users who live with HIV, PLWH: People Living With HIV, HIV: Human Immunodeficiency Virus, BMI: Body Mass Index. Quantitative data with non-normal distribution are presented as median and interquartile range (IQR), and analysis was conducted using ^a^ Kruskal–Wallis’s test with Dunn’s post hoc test. Qualitative data are presented as frequency and percentage; analysis was conducted using ^b^ Fisher’s exact test; *p*-value < 0.05 was considered statistically significant.

**Table 2:** Antiretroviral use and immune status.

	MAHIV	PLWH	*p*
Time of infection (years) ^median, IQR^	6 (3.25,10.0)	6.5 (4.75,12.25)	*0.0907 ^a^*
Time with ART (years) ^median, IQR^	5 (3,10)	6 (4.75,11.25)	*0.3266 ^a^*
Time with actual ART (years) ^median, IQR^	4 (2,5)	4 (2,5)	*0.6494 ^a^*
Viral loads (cell/μL) ^median, IQR^	581 (274, 819.5)	595 (435, 778.5)	*0.4413 ^a^*
Discontinuation of treatment, *n* (%)	25 (62.5%)	16 (38.0%)	*<0.0001 ^b^*

MAHIV: Methamphetamine users who live with HIV, PLWH: People Living With HIV, HIV: Human Immunodeficiency Virus, ART: Antiretroviral Therapy. Quantitative data with non-normal distribution are presented as median and interquartile range (IQR); analysis was conducted using ^a^ Mann-Whitney U test. Qualitative data are presented as frequency and percentage and analyzed with ^b^ Fisher’s exact test; *p*-value < 0.05 was considered statistically significant.

**Table 3:** Current substance use reported by the participant.

	MAHIV	PLWH	Control	*p*
MA, *n* (%)	40 (100%)	0 (0%)	0 (0%)	*<0.0001 ^a^*
Alcohol, *n* (%)	0 (0%)	5 (11.9%)	23 (58.9%)	
Cannabis, *n* (%)	0 (0%)	1 (2.3%)	2 (5.1%)	
Cristal meth, *n* (%)	10 (25%)	1 (2.3%)	0 (0%)	
Poppers, *n* (%)	0 (0%)	4 (9.5%)	0 (0%)	
Combination of 2, *n* (%)	11 (27.5%)	5 (11.9%)	5 (12.8%)	
Combination of more than 2, *n* (%)	16 (40.0%)	3 (7.14%)	0 (0%)	
Substance use according to the urinary drug screening				
Amphetamines, *n* (%)	18 (45%)	0 (0%)	0 (0%)	*<0.0001 ^b^*
MA, *n* (%)	19 (47.5%)	0 (0%)	0 (0%)	*<0.0001 ^b^*
Cannabis, *n* (%)	8 (20%)	0 (0%)	0 (0%)	*0.0001 ^b^*
Cocaine, *n* (%)	2 (5.0%)	0 (0%)	0 (0%)	*0.2095 ^b^*
Benzodiazepines, *n* (%)	1 (2.5%)	0 (0%)	0 (0%)	*0.6529 ^b^*
Opiates, *n* (%)	0 (0%)	0 (0%)	0 (0%)	*1 ^b^*

MA: Methamphetamines, MAHIV: Methamphetamine users that live with HIV, PLWH: People Living With HIV, HIV: Human Immunodeficiency Virus. Qualitative data are presented as frequency and percentage. Differences in categorical variables were assessed using ^a^ Chi-squared test or ^b^ Fisher’s exact test; *p*-value < 0.05 was considered statistically significant.

**Table 4:** Sexual Activity.

	MAHIV	PLWH	Control	*p*
Age of sexual debut ^median, IQR^	16 (13–18)	18 (15–20)	18 (17–19)	*0.0049 ^a^*
Number of sexual partners ^median, IQR^	19.5 (6.25–55.0)	7.5 (4.0–20.0)	5.0 (3.0–10.0)	*<0.0001 ^a^*
Risk sexual practice, *n* (%)	34 (85%)	27 (64.2%)	23 (58.9%)	*0.0241 ^b^*
Monogamous relationship, *n* (%)	21 (52.5%)	28 (66.6%)	37 (94.8%)	*<0.0001 ^b^*
Open relationship, *n* (%)	17 (42.5%)	12 (28.5%)	2 (5.12%)	
Polyamorous relationship, *n* (%)	2 (5.0%)	2 (4.7%)	0 (0%)	
Relations with the same age, *n* (%)	6 (15%)	20 (47%)	21 (53.8%)	*0.0003 ^b^*
Relations with older people, *n* (%)	26 (65%)	18 (42.8%)	9 (23.0%)	
Relation with younger people, *n* (%)	8 (20%)	4 (9.5%)	9 (23.0%)	
Sex with protection, *n* (%)	17 (42.5%)	31 (73.8%)	21 (53.8%)	*0.0134 ^b^*
Sex with no protection, *n* (%)	23 (57.5%)	11 (26.1%)	18 (46.1%)	
History of STD, *n* (%)	33 (82.5%)	26 (61.9%)	5 (12.8%)	*<0.0001 ^b^*
Relations in drugs, *n* (%)	37 (92.5%)	19 (45.2%)	31 (79.4%)	*<0.0001 ^b^*
Casual sexual encounters, *n* (%)	33 (82.5%)	25 (59.5%)	23 (58.9%)	*0.0344 ^b^*
Paid for sex, *n* (%)	7 (17.5%)	0 (0%)	2 (5.1%)	*<0.0001 ^b^*
Had paid for sex, *n* (%)	9 (22.5%)	6 (14.2%)	0 (0%)	
Both, *n* (%)	6 (15.0%)	1 (2.3%)	0 (0%)	
Threesome, *n* (%)	28 (70%)	21 (50%)	8 (20.5%)	*<0.0001 ^b^*
Oral sex without protection, *n* (%)	31 (77.5%)	28 (66.6%)	27 (69.2%)	*0.8194 ^b^*
Oral sex with protection, *n* (%)	5 (12.5%)	9 (21.4%)	8 (20.5%)	
Fisting without protection, *n* (%)	5 (12.5%)	3 (7.14%)	2 (5.12%)	*0.4463 ^b^*
Fisting with protection, *n* (%)	1 (2.5%)	0 (0%)	0 (0%)	

MAHIV: Methamphetamine users who live with HIV, PLWH: People Living with HIV, HIV: Human Immunodeficiency virus. STD: Sexually Transmitted Disease. Quantitative data are presented as median and interquartile range (IQR), and analysis was conducted using ^a^ Kruskal–Wallis’s test with Dunn’s post hoc test. Qualitative data are presented as frequency and percentage and were analyzed with ^b^ Fisher’s exact test. *p*-value < 0.05 was considered statistically significant.

**Table 5:** MoCA cognitive domains.

MoCA	Score	MAHIV	PLWH	Control	*p*
Executive/ Visuospatial Functions Score (5)	01 2 3 4 5	1 (2.5%) 1 (2.5%) 4 (10.0%) 8 (20.0%) 14 (35.0%) 12 (30.0%)	0 (0%) 1 (2.3%) 0 (0%) 12 (28.6%) 17 (40.5%) 12 (28.6%)	0 (0%) 0 (0%) 0 (0%) 3 (7.6%) 25 (64.1%) 11 (28.2%)	*0.0199 ^a^*
Identification and Naming Score (3)	01 2 3	1 (2.5%) 0 (0%) 3 (7.5%) 36 (90.0%)	1 (2.3%) 0 (0%) 2 (4.7%) 39 (93.0%)	0 (0%) 0 (0%) 0 (0%) 39 (100%)	*0.3703 ^a^*
Memory/Delayed Recall Score (5)	01 2 3 4 5	15 (37.5%) 6 (15.0%) 6 (15.0%) 5 (12.5%) 8 (20.0%) 0 (0%)	5 (12%) 5 (12%) 11 (26%) 14 (33.3%) 5 (12%) 2 (4.7%)	0 (0%) 8 (20.5%) 12 (30.7%) 6 (15.4%) 6 (15.4%) 7 (18%)	*<0.0001 ^b^*
Attention and Concentration Score (6)	01 2 3 4 5 6	0 (0%) 2 (5.0%) 4 (10.0%) 7 (17.5%) 9 (22.5%) 11 (27.5%) 7 (17.5%)	0 (0%) 0 (0%) 5 (12%) 7 (16.6%) 9 (21.4%) 11 (26.2%) 10 (23.8%)	0 (0%) 0 (0%) 0 (0%) 2 (5.1%) 5 (13.0%) 14 (35.8%) 18 (46.1%)	*0.0285 ^b^*
Language Score (3)	01 2 3	3 (7.5%) 8 (20.0%) 16 (40.0%) 13 (32.5%)	4 (9.5%) 6 (14.3%) 13 (31.0%) 19 (45.2%)	0 (0%) 2 (5.1%) 12 (30.8%) 25 (64.1%)	*0.0508 ^a^*
Abstraction Score (2)	01 2	8 (20.0%) 9 (22.5%) 23 (57.5%)	4 (9.5%) 7 (16.7) 31 (73.8%)	1 (2.5%) 8 (20.5%) 30 (77.0%)	*0.1239 ^a^*
Orientation Score (6)	0–3 4 5 6	0 (0%) 0 (0%) 3 (7.5%) 37 (92.5%)	0 (0%) 0 (0%) 4 (9.5%) 38 (90.5%)	0 (0%) 1 (2.6%) 3 (7.7%) 35 (89.7%)	*0.9194 ^a^*
MoCA Interpretation					
	**MAHIV**		**PLWH**	**Control**	***p***
No cognitive impairment, *n* (%)	4 (10.0%)		7 (16.7%)	13 (33.3%)	*0.0007 ^a^*
Mild cognitive impairment, *n* (%)	20 (50.0%)		25 (59.5%)	20 (51.3%)	
Cognitive impairment, *n* (%)	13 (32.5%)		7 (16.7%)	0 (0%)	

MAHIV: Methamphetamine users who live with HIV, PLWH: People living with HIV, HIV: Human Immunodeficiency virus, MoCA: Montreal Cognitive Assessment. Differences in categorical variables were assessed using ^a^ Fisher’s exact test or ^b^ Chi-squared test; *p*-value < 0.05 was considered statistically significant.

**Table 6:** Beck Depression Inventory–II (BDI-II).

BDI-II Interpretation	MAHIV	PLWH	Control	*p*
No depressive disorder, *n* (%)	12 (30.0%)	26 (61.9%)	24 (61.5%)	*0.0046 ^a^*
Minimal depression, *n* (%)	10 (25.0%)	11 (26.1%)	10 (25.6%)	
Low depression, *n* (%)	13 (32.5%)	5 (12.0%)	4 (10.3%)	
Moderate depression, *n* (%)	5 (12.5%)	0 (0%)	1 (2.6%)	
Severe depression, *n* (%)	0 (0%)	0 (0%)	0 (0%)	

MAHIV: Methamphetamine users who live with HIV, PLWH: People living with HIV, HIV: Human Immunodeficiency virus, BDI-II: Beck Depression Inventory. Qualitative data were analyzed with ^a^ Fisher’s test. *p*-value < 0.05 was considered statistically significant.

**Table 7:** Sleep quality assessment (PSQI).

PSQI	Score	MAHIV	PLWH	Control	*p*
Component 1: subjective sleep quality, *n* (%)	01 2 3	14 (35%) 16 (40%) 8 (20%) 2 (5.0%)	17 (40.4%) 18 (42.8%) 7 (16.6%) 0 (0%)	12 (30.7%) 18 (46.1%) 8 (20.5%) 1 (2.5%)	*0.8692 ^a^*
Component 2: Sleep latency, *n* (%)	01 2 3	9 (22.5%) 13 (32.5%) 11 (27.5%) 7 (17.5%)	14 (33.5%) 11 (26.1%) 11 (26.1%) 6 (14.2%)	12 (30.7%) 19 (48.7%) 7 (17.9%) 1 (2.5%)	*0.1495 ^a^*
Component 3: Sleep duration, *n* (%)	01 2 3	23 (57.5%) 4 (10.0%) 6 (15.0%) 7 (17.5%)	18 (42.8%) 14 (33.3%) 6 (14.3%) 4 (9.5%)	2 (5.1%) 10 (25.6%) 17 (42.5%) 10 (25.6%)	*<0.0001 ^b^*
Component 4: Efficacy of usual sleep, *n* (%)	01 2 3	19 (47.5%) 8 (20.0%) 8 (20.0%) 5 (12.5%)	28 (66.6%) 4 (9.5%) 6 (14.2%) 4 (9.5%)	20 (5.1%) 14 (35.8%) 2 (5.1%) 3 (7.6%)	*0.0596 ^a^*
Component 5: Sleep disturbances, *n* (%)	01 2 3	1 (2.5%) 26 (65.0%) 12 (30.0%) 1 (2.5%)	3 (7.1%) 30 (71.4%) 8 (19.0%) 1 (2.3%)	2 (5.1%) 28 (71.7%) 8 (20.5%) 1 (2.5%)	*0.9102 ^a^*
Component 6: Use of hypnotic medication, *n* (%)	01 2 3	27 (67.5%) 1 (2.5%) 5 (12.5%) 7 (17.5%)	35 (83.3%) 2 (4.7%) 1 (2.3%) 4 (9.5%)	31 (79.4%) 5 (12.8%) 2 (5.1%) 1 (2.5%)	*0.0681 ^a^*
Component 7: Daytime dysfunction, *n* (%)	01 2 3	12 (30.0%) 15 (37.5%) 9 (22.5%) 4 (10.0%)	17 (40.4%) 20 (47.6%) 5 (11.9%) 0 (0%)	5 (12.8%) 17 (43.5%) 15 (38.4%) 2 (5.1%)	*0.0083 ^a^*

MAHIV: Methamphetamine users who live with HIV, PLWH: People Living with HIV, HIV: Human Immunodeficiency virus, PSQI: Pittsburgh Sleep Quality Index. Differences in categorical variables were assessed using ^a^ Fisher’s exact test or ^b^ Chi-squared test; *p*-value < 0.05 was considered statistically significant.

**Table 8:** Correlation between blood sCD14 and MoCA, GAD-7, BDI-II, and PSQI scores.

	MoCA	GAD-7	BDI-II	PSQI
sCD14	−0.2420 ^a^**	0.3325 ^a^***	0.2551 ^a^**	0.1507 ^a^

sCD14: soluble CD14, MoCA: Montreal Cognitive Assessment, GAD-7: Generalized Anxiety Disorder-7 item scale, BDI-II: Beck Depression Inventory, PSQI: Pittsburgh Sleep Quality Index. ^a^ Spearman Correlation test. *p*-value: **: <0.005, ***: <0.0005.

**Table 9:** Multivariable regression analysis for demographic variables (age, education, and sexual orientation).

Outcome	β (Group)	SE	*p*
MoCA Punctuation	1.78	0.44	*<0.001*
GAD-7 Punctuation	−2.53	0.69	*<0.001*
BDI-II Punctuation	−3.32	0.75	*<0.001*
sCD14	−164,000	58,000	*0.006*
NSE	0.048	0.017	*0.007*

MoCA: Montreal Cognitive Assessment, GAD-7: Generalized Anxiety Disorder-7 item scale, BDI-II: Beck Depression Inventory. β coefficients correspond to the adjusted effect of group on each outcome. All multivariable regression models were adjusted for age, education, and sexual orientation.

## Data Availability

The data presented in this study are available on request from the corresponding author for privacy reasons.

## References

[1] UNODC. World Drug Report 2024 (United Nations Publication, 2024). https://www.unodc.org/unodc/en/data-and-analysis/world-drug-report-2024.html.

[2] Carrico A.W., Horvath K.J., Grov C., Moskowitz J.T., Pahwa S., Pallikkuth S., Hirshfield S. (2020). Double Jeopardy: Methamphetamine Use and HIV as Risk Factors for COVID-19. AIDS Behav..

[3] Niu M., Morsey B., Lamberty B.G., Emanuel K., Yu F., León-Rivera R., Berman J.W., Gaskill P.J., Matt S.M., Ciborowski P.S. (2020). Methamphetamine Increases the Proportion of SIV-Infected Microglia/Macrophages, Alters Metabolic Pathways, and Elevates Cell Death Pathways: A Single-Cell Analysis. Viruses.

[4] Hoenigl M., Chaillon A., Moore D.J., Morris S.R., Smith D.M., Little S.J. (2016). Clear Links Between Starting Methamphetamine and Increasing Sexual Risk Behavior. JAIDS J. Acquir. Immune Defic. Syndr..

[5] Lai H.-H., Wang C.-C., Yen T.-F., Yeh P.-T., Yen Y.-F., Hsu S.-H. (2024). Antiretroviral Treatment Adherence among People Living with HIV in Taipei, Taiwan. J. Epidemiol. Glob. Health.

[6] Lai H.-H., Kuo Y.-C., Kuo C.-J., Lai Y.-J., Chen M., Chen Y.-T., Chen C.-C., Yen M.-Y., Hu B.-S., Wang T.-H. (2020). Methamphetamine Use Associated with Non-Adherence to Antiretroviral Treatment in Men Who Have Sex with Men. Sci. Rep..

[7] Cysique L.A., Brew B.J. (2019). Vascular Cognitive Impairment and HIV-Associated Neurocognitive Disorder: A New Paradigm. J. Neurovirol..

[8] Prakoeswa F.R.S., Maharani F., Bestari R.S., Aisyah R., Ichsan B., Nursanto D., Listiansyah R., Tuanaya M.R.N. (2025). Aging and HIV: Recent Findings in Contributing Factors. AIDS Res. Treat..

[9] Thompson L.J.-P., Genovese J., Hong Z., Singh M.V., Singh V.B. (2024). HIV-Associated Neurocognitive Disorder: A Look into Cellular and Molecular Pathology. Int. J. Mol. Sci..

[10] Swanta N., Aryal S., Nejtek V., Shenoy S., Ghorpade A., Borgmann K. (2020). Blood-Based Inflammation Biomarkers of Neurocognitive Impairment in People Living with HIV. J. Neurovirol..

[11] Sonti S., Tyagi K., Pande A., Daniel R., Sharma A.L., Tyagi M. (2022). Crossroads of Drug Abuse and HIV Infection: Neurotoxicity and CNS Reservoir. Vaccines.

[12] Yang X., Wang Y., Li Q., Zhong Y., Chen L., Du Y., He J., Liao L., Xiong K., Yi C. (2018). The Main Molecular Mechanisms Underlying Methamphetamine- Induced Neurotoxicity and Implications for Pharmacological Treatment. Front. Mol. Neurosci..

[13] Moratalla R., Ares-Santos S., Granado N. (2014). Neurotoxicity of Methamphetamine. Handbook of Neurotoxicity.

[14] Cornea A., Lata I., Simu M., Rosca E.C. (2023). Assessment and Diagnosis of HIV-Associated Dementia. Viruses.

[15] Letendre S. (2011). Central Nervous System Complications in HIV Disease: HIV-Associated Neurocognitive Disorder. Top. Antivir. Med..

[16] Chilunda V., Calderon T.M., Martinez-Aguado P., Berman J.W. (2019). The Impact of Substance Abuse on HIV-Mediated Neuropathogenesis in the Current ART Era. Brain Res..

[17] Mediouni S., Garibaldi Marcondes M.C., Miller C., McLaughlin J.P., Valente S.T. (2015). The Cross-Talk of HIV-1 Tat and Methamphetamine in HIV-Associated Neurocognitive Disorders. Front. Microbiol..

[18] Alvarez-Carbonell D., Ye F., Ramanath N., Garcia-Mesa Y., Knapp P.E., Hauser K.F., Karn J. (2019). Cross-Talk between Microglia and Neurons Regulates HIV Latency. PLoS Pathog..

[19] Fattakhov N., Torices S., Stangis M., Park M., Toborek M. (2021). Synergistic Impairment of the Neurovascular Unit by HIV-1 Infection and Methamphetamine Use: Implications for HIV-1-Associated Neurocognitive Disorders. Viruses.

[20] Carrico A.W., Hunt P.W., Neilands T.B., Dilworth S.E., Martin J.N., Deeks S.G., Riley E.D. (2019). Stimulant Use and Viral Suppression in the Era of Universal Antiretroviral Therapy. JAIDS J. Acquir. Immune Defic. Syndr..

[21] Roy R.J., Parvaz M.A., Wakabayashi K.T., Blair R.J.R., Hubbard N.A. (2024). Methamphetamine-related Working Memory Difficulties Underpinned by Reduced Frontoparietal Responses. Addict. Biol..

[22] Mustafa A.I., Woods S.P., Loft S., Morgan E.E. (2023). Lower Prospective Memory Is Associated with Higher Neurocognitive Dispersion in Two Samples of People with HIV: A Conceptual Replication Study. J. Int. Neuropsychol. Soc..

[23] Poquette A.J., Moore D.J., Gouaux B., Morgan E.E., Grant I., Woods S.P. (2013). Prospective Memory and Antiretroviral Medication Non-Adherence in HIV: An Analysis of Ongoing Task Delay Length Using the Memory for Intentions Screening Test. J. Int. Neuropsychol. Soc..

[24] Carrico A.W., Cherenack E.M., Roach M.E., Riley E.D., Oni O., Dilworth S.E., Shoptaw S., Hunt P., Roy S., Pallikkuth S. (2018). Substance-Associated Elevations in Monocyte Activation among Methamphetamine Users with Treated HIV Infection. AIDS.

[25] Zhu Z., Xu J., Jin Y., Wang L., Li X. (2022). Effects of Aerobic Exercise on Markers of Brain Injury in Methamphetamine-Dependent Individuals: A Randomized Controlled Trial. Brain Sci..

[26] Lodge W., Kelly P.J.A., Napoleon S., Plezia S., Mimiaga M.J., Biello K.B. (2024). Prevalence of Methamphetamine Use among Gay, Bisexual and Other Men Who Have Sex with Men: A Systematic Review and Meta-Analysis. Int. J. Drug Policy.

[27] Eustaquio P.C., Smyth J., Salisi J.A. (2024). The Risks for HIV and Sexually Transmitted Infections Among Men Who Have Sex with Men Who Engage in Chemsex in Low- and Middle-Income Countries: A Mixed Methods Systematic Review and Meta-Analysis. AIDS Behav..

[28] De La Mora L., Laguno M., Torres B., Chivite I., Foncillas A., Inciarte A., Calvo J., González-Cordón A., Ambrosioni J., Berrocal L. (2025). Long-Term Health Outcomes of People with HIV Engaged in Chemsex: A Prospective Cohort Study on Drug Use, Sexual Behaviour, Sexually-Transmitted Infections and Vulnerability. Infect. Dis. Ther..

[29] Pessina R., PavanelloDecaro S., Torri C., Prunas A. (2025). Chemsex and Psychosexual Health in a Large Italian Sample of Men Who Have Sex with Men (MSM). Sex. Cult..

[30] Jones C.M., Compton W.M., Mustaquim D. (2020). Patterns and Characteristics of Methamphetamine Use Among Adults—United States, 2015–2018. MMWR Morb. Mortal. Wkly. Rep..

[31] Necho M., Zenebe Y., Tiruneh C., Ayano G., Yimam B. (2022). The Global Landscape of the Burden of Depressive Symptoms/Major Depression in Individuals Living With HIV/AIDs and Its Effect on Antiretroviral Medication Adherence: An Umbrella Review. Front. Psychiatry.

[32] Remien R.H., Stirratt M.J., Nguyen N., Robbins R.N., Pala A.N., Mellins C.A. (2019). Mental Health and HIV/AIDS. AIDS.

[33] Sin N.L., DiMatteo M.R. (2014). Depression Treatment Enhances Adherence to Antiretroviral Therapy: A Meta-Analysis. Ann. Behav. Med..

[34] Rosen A.D., Javanbakht M., Shoptaw S.J., Seamans M.J., Gorbach P.M. (2024). Associations of Sleep Deficiency with Sexual Risk Behaviors and HIV Treatment Outcomes Among Men Who Have Sex with Men Living with or at High Risk of Acquiring HIV. JAIDS J. Acquir. Immune Defic. Syndr..

[35] García A., Del Angel J., Borrani J., Ramirez C., Valdez P. (2021). Sleep Deprivation Effects on Basic Cognitive Processes: Which Components of Attention, Working Memory, and Executive Functions Are More Susceptible to the Lack of Sleep?. Sleep Sci..

[36] Gruber R., Gauthier-Gagné G., Little C., Fu Z. (2023). The Associations between the Homeostatic and Circadian Sleep Processes and the Neurobehavioral Functioning (NBF) of Individuals with ADHD—A Systematic Review. Brain Sci..

[37] Hyndych A., El-Abassi R., Mader E.C. (2025). The Role of Sleep and the Effects of Sleep Loss on Cognitive, Affective, and Behavioral Processes. Cureus.

[38] Sun-Suslow N., Saloner R., Serrano V., Umlauf A., Morgan E.E., Ellis R.J., Letendre S., Grant I., Heaton R.K. (2020). Lifetime Methamphetamine Use Disorder and Reported Sleep Quality in Adults Living with HIV. AIDS Behav..

[39] Przybyla S., Ashare R.L., Cioffi L., Plotnik I., Shuter J., Seng E.K., Weinberger A.H. (2022). Substance Use and Adherence to Antiretroviral Therapy among People Living with HIV in the United States. Trop. Med. Infect. Dis..

[40] Martinez E., Dorfman D. (2024). Addressing Methamphetamine Use in Persons with HIV. AIDS.

[41] Khan R., Turner A., Berk M., Walder K., Rossell S., Guerin A.A., Kim J.H. (2025). Genes, Cognition, and Their Interplay in Methamphetamine Use Disorder. Biomolecules.

[42] Love S., Nicolls M., Rowland B., Davey J. (2024). The Impact of Methamphetamine Use and Dependence: A Systematic Review on the Cognitive-Behavioural Implications for Road Safety. Transp. Res. Part F Traffic Psychol. Behav..

[43] Basterfield C., Hester R., Bowden S.C. (2019). A Meta-Analysis of the Relationship between Abstinence and Neuropsychological Functioning in Methamphetamine Use Disorder. Neuropsychology.

[44] Simon S.L., Dean A.C., Cordova X., Monterosso J.R., London E.D. (2010). Methamphetamine Dependence and Neuropsychological Functioning: Evaluating Change During Early Abstinence. J. Stud. Alcohol Drugs.

[45] Rogers J.M., Iudicello J.E., Marcondes M.C.G., Morgan E.E., Cherner M., Ellis R.J., Letendre S.L., Heaton R.K., Grant I. (2023). The Combined Effects of Cannabis, Methamphetamine, and HIV on Neurocognition. Viruses.

[46] Babkina A.S., Lyubomudrov M.A., Golubev M.A., Pisarev M.V., Golubev A.M. (2024). Neuron-Specific Enolase—What Are We Measuring?. Int. J. Mol. Sci..

[47] Benedict C., Cedernaes J., Giedraitis V., Nilsson E.K., Hogenkamp P.S., Vågesjö E., Massena S., Pettersson U., Christoffersson G., Phillipson M. (2014). Acute Sleep Deprivation Increases Serum Levels of Neuron-Specific Enolase (NSE) and S100 Calcium Binding Protein B (S-100B) in Healthy Young Men. Sleep.

[48] Ellis R.J., Marquine M.J., Kaul M., Fields J.A., Schlachetzki J.C.M. (2023). Mechanisms Underlying HIV-Associated Cognitive Impairment and Emerging Therapies for Its Management. Nat. Rev. Neurol..

[49] Miao L., Wang H., Li Y., Huang J., Wang C., Teng H., Xu L., Yang X., Tian Y., Yang G. (2024). Mechanisms and Treatments of Methamphetamine and HIV-1 Co-Induced Neurotoxicity: A Systematic Review. Front. Immunol..

[50] Rogers J.M., Chentsova V.O., Wang C.X., Marcondes M.C.G., Cherner M., Ellis R.J., Letendre S.L., Heaton R.K., Grant I., Iudicello J.E. (2025). Cannabis Use Moderates Methamphetamine- and HIV-Related Inflammation: Evidence from Human Plasma Markers. Viruses.

[51] Singh M.V., Uddin M.N., CovacevichVidalle M., Sutton K.R., Boodoo Z.D., Peterson A.N., Tyrell A., Tivarus M.E., Wang H.Z., Sahin B. (2024). Non-Classical Monocyte Levels Correlate Negatively with HIV-Associated Cerebral Small Vessel Disease and Cognitive Performance. Front. Cell. Infect. Microbiol..

[52] Lyons J.L., Uno H., Ancuta P., Kamat A., Moore D.J., Singer E.J., Morgello S., Gabuzda D. (2011). Plasma SCD14 Is a Biomarker Associated With Impaired Neurocognitive Test Performance in Attention and Learning Domains in HIV Infection. JAIDS J. Acquir. Immune Defic. Syndr..

[53] Moschopoulos C.D., Stanitsa E., Protopapas K., Vatsi A., Galani I., Zetterberg H., Beratis I., Fragkou P.C., Tsiodras S., Kavatha D. (2025). Prospective Neuropsychological and Plasma Biomarker Changes in Treatment-Naïve People Living with HIV After Antiretroviral Treatment Initiation. Biomedicines.

[54] Jumare J., Akolo C., Ndembi N., Bwala S., Alabi P., Okwuasaba K., Adebiyi R., Umlauf A., Cherner M., Abimiku A. (2020). Elevated Plasma Levels of SCD14 and MCP-1 Are Associated With HIV Associated Neurocognitive Disorders Among Antiretroviral-Naive Individuals in Nigeria. JAIDS J. Acquir. Immune Defic. Syndr..

[55] Demirci E., Tastepe N., Gul M.K., Ozmen S., Kilic E. (2023). S100B and Neuron-Specific Enolase Levels as Brain Injury Biomarkers in Internet Addiction: Effect of Sleep. Pediatr. Neurol..

[56] Acuña A.M., Nagy E.K., Legg J.L., Rodarte S.E., Olive M.F. (2025). Characterization of Serum and Brain Cytokine Levels Following Prolonged Binge-like Methamphetamine Self-Administration and Cued Methamphetamine Seeking. J. Neuroimmunol..

[57] Pang L., Wang Y. (2023). Overview of Blood-Brain Barrier Dysfunction in Methamphetamine Abuse. Biomed. Pharmacother..

[58] Northrop N.A. (2015). Methamphetamine Effects on Blood-Brain Barrier Structure and Function. Front. Neurosci..

[59] Stewart J.C., Polanka B.M., So-Armah K.A., White J.R., Gupta S.K., Kundu S., Chang C.-C.H., Freiberg M.S. (2020). Associations of Total, Cognitive/Affective, and Somatic Depressive Symptoms and Antidepressant Use with Cardiovascular Disease–Relevant Biomarkers in HIV: Veterans Aging Cohort Study. Psychosom. Med..

[60] Anderson A.M., Ma Q., Letendre S.L., Iudicello J. (2021). Soluble Biomarkers of Cognition and Depression in Adults with HIV Infection in the Combination Therapy Era. Curr. HIV/AIDS Rep..

[61] Madison A.A., Andridge R., Padin A.C., Wilson S., Bailey M.T., Alfano C.M., Povoski S.P., Lipari A.M., Agnese D.M., Carson W.E. (2020). Endotoxemia Coupled with Heightened Inflammation Predicts Future Depressive Symptoms. Psychoneuroendocrinology.

[62] Morena D., Lippi M., Scopetti M., Turillazzi E., Fineschi V. (2025). Leaky Gut Biomarkers as Predictors of Depression and Suicidal Risk: A Systematic Review and Meta-Analysis. Diagnostics.

[63] Mudra Rakshasa-Loots A. (2023). Depression and HIV: A Scoping Review in Search of Neuroimmune Biomarkers. Brain Commun..

[64] Mudra Rakshasa-Loots A., Bakewell N., Sharp D.J., Gisslén M., Zetterberg H., Alagaratnam J., Wit F.W.N.M., Kootstra N.A., Winston A., Reiss P. (2023). Biomarkers of Central and Peripheral Inflammation Mediate the Association between HIV and Depressive Symptoms. Transl. Psychiatry.

[65] Cherenack E.M., Chavez J.V., Martinez C., Hirshfield S., Balise R., Horvath K.J., Viamonte M., Jimenez D.E., Paul R., Dilworth S.E. (2023). Stimulant Use, HIV, and Immune Dysregulation among Sexual Minority Men. Drug Alcohol Depend..

[66] Hmiel L., Zhang S., Obare L.M., Santana M.A.d.O., Wanjalla C.N., Titanji B.K., Hileman C.O., Bagchi S. (2024). Inflammatory and Immune Mechanisms for Atherosclerotic Cardiovascular Disease in HIV. Int. J. Mol. Sci..

[67] Merino J.J., Parmigiani-Cabaña J.M., Parmigiani-Izquierdo J.M., Fernández-García R., Cabaña-Muñoz M.E. (2024). Decreased Systemic Monocyte Colony Protein-1 (MCP-1) Levels and Reduced SCD14 Levels in Curcumin-Treated Patients with Moderate Anxiety: A Pilot Study. Antioxidants.

[68] Milaneschi Y., Kappelmann N., Ye Z., Lamers F., Moser S., Jones P.B., Burgess S., Penninx B.W.J.H., Khandaker G.M. (2021). Association of Inflammation with Depression and Anxiety: Evidence for Symptom-Specificity and Potential Causality from UK Biobank and NESDA Cohorts. Mol. Psychiatry.

[69] Kidd J.D., Alves J., Mukherjee R., Whitley S.D., Wiegand T.J., Stancliff S.L., Norton B., Ellendon N., Hoffmann C.J., Gonzalez C.J. (2025). Chemsex: Questions and Answers. Substance Use Guidelines Committee. https://www.hivguidelines.org/guideline/chemsex/.

[70] Blair C.S., Shoptaw S.J. (2024). Managing Stimulant Use Among People with HIV: Harm-Reduction Strategies From Behavior to Medication. Top. Antivir. Med..

[71] Gandhi R.T., Landovitz R.J., Sax P.E., Smith D.M., Springer S.A., Günthard H.F., Thompson M.A., Bedimo R.J., Benson C.A., Buchbinder S.P. (2025). Antiretroviral Drugs for Treatment and Prevention of HIV in Adults: 2024 Recommendations of the International Antiviral Society–USA Panel. JAMA.

[72] Parsons J.T., John S.A., Millar B.M., Starks T.J. (2018). Testing the Efficacy of Combined Motivational Interviewing and Cognitive Behavioral Skills Training to Reduce Methamphetamine Use and Improve HIV Medication Adherence Among HIV-Positive Gay and Bisexual Men. AIDS Behav..

[73] Glasner S., Patrick K., Ybarra M., Reback C.J., Ang A., Kalichman S., Bachrach K., Garneau H.C., Venegas A., Rawson R.A. (2022). Promising Outcomes from a Cognitive Behavioral Therapy Text-Messaging Intervention Targeting Drug Use, Antiretroviral Therapy Adherence, and HIV Risk Behaviors among Adults Living with HIV and Substance Use Disorders. Drug Alcohol Depend..

[74] Sumari-de Boer I.M., Ngowi K.M., Sonda T.B., Pima F.M., Masika B.L.V., Sprangers M.A.G., Reiss P., Mmbaga B.T., Nieuwkerk P.T., Aarnoutse R.E. (2021). Effect of Digital Adherence Tools on Adherence to Antiretroviral Treatment Among Adults Living with HIV in Kilimanjaro, Tanzania: A Randomized Controlled Trial. JAIDS J. Acquir. Immune Defic. Syndr..

[75] Ukoaka B.M., Ugwuanyi E.A., Ukueku K.O., Ajah K.U., Udam N.G., Daniel F.M., Wali T.A., Gbuchie M.A. (2024). Digital Tools for Improving Antiretroviral Adherence among People Living with HIV in Africa. J. Med. Surg. Public Health.

[76] Msosa T.C., Phuka F., Sumari-de Boer M., Mhango O., Twabi H.H., Mukoka M., Swai I., Aarnoutse R., de Wit T.F.R., Ngowi K. (2025). The Acceptability of a Real-Time Medication Monitoring-Based Digital Adherence Tool Among Young People Living with HIV in Malawi. AIDS Behav..

[77] Wong N.S., Chung S.L., Lee K.C.-K., LEE S.-S. (2025). Interrelationship between Chemsex Engagement and PrEP (Pre-Exposure Prophylaxis) Experience in Men Who Have Sex with Men: A Community-Based Cross-Sectional Study. Sex. Transm. Infect..

[78] Del Pozo-Herce P., Martínez-Sabater A., Sanchez-Palomares P., Garcia-Boaventura P.C., Chover-Sierra E., Martínez-Pascual R., Gea-Caballero V., Saus-Ortega C., Ballestar-Tarín M.L., Karniej P. (2024). Effectiveness of Harm Reduction Interventions in Chemsex: A Systematic Review. Healthcare.

